# Supplementary material for: Nano-spectroscopy of excitons in atomically thin transition metal dichalcogenides
Source: Nat Commun. 2022 Jan 27;13:542. doi: 10.1038/s41467-022-28117-x (PMC8795359; doi:10.1038/s41467-022-28117-x)
Supplement: Supplementary file 1 — Supplementary Information [file 41467_2022_28117_MOESM1_ESM.pdf]

Supplementary Information

Nano-spectroscopy of excitons in atomically thin transition metal dichalcogenides

Shuai Zhang, Baichang Li, Xinzhong Chen, Francesco L. Ruta, Yinming Shao, Aaron J. Sternbach, A. S. McLeod, Zhiyuan Sun, Lin Xiong, S. L. Moore, Xinyi Xu, Wenjing Wu, Sara Shabani, Lin Zhou, Zhiying Wang, Fabian Mooshammer, Essance Ray, Nathan Wilson, P.J Schuck, C. R. Dean, A. N. Pasupathy, Michal Lipson, Xiaodong Xu, Xiaoyang Zhu, A. J. Millis, Mengkun Liu, James C. Hone, D.N. Basov*

* Corresponding email: db3056@columbia.edu

This file includes:

Supplementary Note

Supplementary Figures. 1-7

References

Supplementary Note 1: The analytical relation between the near-field signal and the permittivity

In this section, we derive a simplified, yet intuitive relation between the scattering response and the permittivity of the TMDs before performing the numerical simulation in the next section. It should be noted that all the data analyses in the manuscript are based on rigorous numerical simulation discussed in the next section. The sample structure includes three regions: air (denoted by subscript "a") at the top, TMDs with thickness d in the middle, and semi-infinite substrate (denoted by subscript "s").

The Fresnel reflection coefficient for p-polarized electric field servers as an estimate for the scattering response in this section and is given by

$$r_p = \frac{\frac{\varepsilon_s^t}{k_s^z} - \frac{\varepsilon_a^t}{k_a^z} + \frac{\sigma_s}{\omega}}{\frac{\varepsilon_s^t}{k_s^z} + \frac{\varepsilon_a^t}{k_a^z} + \frac{\sigma_s}{\omega}} \quad (1)$$

Here, $\sigma_s = -id\varepsilon_0(\varepsilon_r - 1)\omega$ is the sheet conductivity of van der Waals TMDs, where ε_0 is vacuum permittivity, ε_r is the relative permittivity of TMDs. For atomically thin TMDS, only the in-plane conductivity contributes to the reflectivity[1]. For other materials, to take the anisotropy of materials into consideration, we use both in-plane and out-of-plane permittivity. $\varepsilon_s^t, \varepsilon_a^t$ are the in-plane permittivities of the substrate and air, respectively. $\varepsilon_s^z, \varepsilon_a^z$ are the out-of-plane permittivities of the substrate BN and air, respectively. $\varepsilon_j = \sqrt{\varepsilon_j^t \cdot \varepsilon_j^z}$. For air, $\varepsilon_a^z = \varepsilon_a^t = \varepsilon_a$; for the h-BN, the in-plane and our-of-plane permittivity do not have much difference in the spectral range we investigated, so we assume $\varepsilon_s^z = \varepsilon_s^t =$

ε_s. k_j^z (j corresponds to air or substrate) is the wave vector along out-of-plane direction, which is given by $k_j^z = \sqrt{\varepsilon_j^t \left(\frac{\omega^2}{c^2} - \frac{q^2}{\varepsilon_j^z}\right)}$, where q is the in-plane wave vector.

At large q, which is determined by the tip radius, we can make the approximation, $k_j^z \approx -iq$.

From these definition, we can get,

$$r_p = \frac{\varepsilon_s - \varepsilon_a + i\frac{\sigma}{\omega}q}{\varepsilon_s + \varepsilon_a + i\frac{\sigma}{\omega}q} \qquad (2)$$

We denote Δr_p as the difference in reflection coefficients for structures including and excluding the TMDs.

$$\Delta r_p = \frac{\varepsilon_s - \varepsilon_a + i\frac{\sigma}{\omega}q}{\varepsilon_s + \varepsilon_a + i\frac{\sigma}{\omega}q} - \frac{\varepsilon_s - \varepsilon_a}{\varepsilon_s + \varepsilon_a} = \frac{\varepsilon_s - \varepsilon_a}{\varepsilon_s + \varepsilon_a}\left(\frac{i2\varepsilon_a \sigma q/\omega}{(\varepsilon_s - \varepsilon_a)(\varepsilon_s + \varepsilon_a) + \frac{i(\varepsilon_s - \varepsilon_a)\sigma q}{\omega}}\right) \qquad (3)$$

Here, the term $\frac{i\sigma q}{\omega}$ encodes the properties of the TMDs and determines the near-field signal.

Using $\frac{i2\varepsilon_a \sigma q}{\omega} = 2\varepsilon_0^2 \varepsilon_{ar}(\varepsilon_r - 1)dq$ and $r_0 = \frac{\varepsilon_s - \varepsilon_a}{\varepsilon_s + \varepsilon_a}$, where ε_r and ε_{ar} are the relative permittivity of the TMDs and the air, respectively. We finally obtain,

$$\Delta r_p = r_0 \left(\frac{2\varepsilon_{ar}(\varepsilon_r - 1)dq}{(\varepsilon_{sr} - \varepsilon_{ar})(\varepsilon_{sr} + \varepsilon_{ar}) + (\varepsilon_{sr} - \varepsilon_{ar})(\varepsilon_r - 1)dq}\right) \qquad (4)$$

This is the key result of this part.

At the far-field limit and in the near-infrared spectral range, the large damping at the room temperature makes that the real dielectric function remains positive and the

imaginary dielectric function has a finite positive value. With a layer thickness of 0.7 nm, this yields $\varepsilon_{ar} d(\varepsilon_r - 1)q \ll 1$.

At the near-field limit, q is determined by the tip apex. The dominant momentum is about $1/R$, where R is the tip radius (~25 nm). When the thickness of the material is smaller than the tip radius, $(\varepsilon_{sr} - \varepsilon_{ar})(\varepsilon_r - 1)dq$ remains marginal compared to $(\varepsilon_{sr} - \varepsilon_{ar})$ $(\varepsilon_{sr} + \varepsilon_{ar})$. We can evaluate the two terms in the denominator using ε_{sr} = 3.8, ε_{ar} = 1, the terms $(\varepsilon_{sr} - \varepsilon_{ar})(\varepsilon_{sr} + \varepsilon_{ar})$ is about 16.24, while the second term $(\varepsilon_{sr} - \varepsilon_{ar})(\varepsilon_r - 1)dq$ is about 0.8. Here we take d = 0.7 nm, q = $4 \times 10^5\ cm^{-1}$, and $\varepsilon_r =$ 10.

From the above discussion, we find that in the few-layer TMD case,

$$\Delta r_p \approx r_0 \left(\frac{2\varepsilon_{ar}(\varepsilon_r - 1)dq}{(\varepsilon_{sr} - \varepsilon_{ar})(\varepsilon_{sr} + \varepsilon_{ar})} \right) \qquad (5)$$

Here, r_0 is the complex reflection without TMDs. From the formula, we can clearly see that Δr_p is proportional to TMD's permittivity.

The near-field signal can be denoted as[2,3],

$$s \approx \int W(q) r_p dq \qquad (6)$$

where the weighting function $W(q)$ denotes the momentum q distribution and is determined by the tip geometry. Here all the data can be normalized to the substrate (h-BN). In weak resonance limit, r_p of the substrate and the TMDs do not have much dependence on the q.

$$s_{normalized} = \frac{s_{TMDs} e^{i\varphi_{TMDs}}}{s_{BN} e^{i\varphi_{BN}}}$$

$$= \frac{\int \omega(k)_{Tip} r_{p_{TMDs}} dk}{\int \omega(k)_{Tip} r_{p_{BN}} dk} \approx \frac{r_{p_{TMDs}} \int W(q)_{Tip} dk}{r_{p_{BN}} \int W(q)_{Tip} dk} \propto \frac{\Delta r_p}{r_0} \qquad (7)$$

Therefore, the normalized near-field signal is approximately proportional to Δr_p.

The key results derived from this formula can be summarized as follows:

1) When the excitation energy is far away from the excitonic resonance energies, the imaginary part of permittivity is zero. So the phase signal for the sample with and without TMDs are the same. Generally, the phase signal peaks around the exciton resonance energy, where the imaginary part of the permittivity is maximum.
2) The near field signal is proportional to the layer thickness if the change of the permittivity is negligible. So the near field amplitude linearly increases as a function of the layer numbers (see Supplementary Figure 7).
3) The reflectivity is proportional to the momentum q. Therefore, the large momentum given by the sharp tip in the near-field experiment boosts the light-TMDs interaction. This is one of the factors that give raise to strong near-field interaction at the nanometer scale.

We note that in this section we focus on the case that the dielectric function has large damping. When damping is reduced, such as through low-temperature experiments, the real dielectric function will go across zero and r_p will strongly depend on the momentum q. These consequences give rise to different relation between the near-field signal and the dielectric function.

Supplementary Note 2: Point dipole model of s-SNOM scattering amplitude and phase

The point dipole model[3] is used to interpret the data as it is well documented to capture the response of atomically thin samples transferred on thick substrates[3,4]. The short detection depth in our experiment also justifies the point dipole as a good model description of our experiment. The near-field signals are almost the same across regions with different h-BN thickness, 30 and 40 nm (Fig. 2 and Supplementary Figure 3). This negligible contrast indicates that the near-field detection depth is no more than 30 nm, consistent with the recent simulation results, which show that the high harmonics demodulation and the short wavelength reduce the s-SNOM detection depth[5,6]. Furthermore, recently both the transverse electric (TE) and transverse magnetic (TM) waveguide modes were observed in TMDs flakes by s-SNOM at NIR frequency, which is in contrast to mid-IR experiments where only the TM model can be detected[7]. The observation of the TE model at the NIR frequency further confirms that the tip effective length is comparable to its horizontal scale, and thus the tip can be treated as a point dipole.

Thus, the calculation is performed using the well-established point-dipole model framework for multi-layered sample[3], where the AFM tip is approximated by a point dipole located at its apex. The tip radius is $a = 25\,nm$. The harmonic motion of the tip can be described by $z(t) = h_0 + A(1 - \cos(\Omega t))$, where $A = 50\,nm$ is the tip tapping amplitude and Ω is the tip tapping frequency. h_0 is set to be $23\,nm$. The near-field interaction of the tip and the sample manifest itself via the modified dipole moment $\boldsymbol{p}$, which is directly proportional to the far-field scattering. The complex-valued scattered field demodulated at n-th harmonics of Ω is thus given by

$$s_n e^{i\phi_n} \propto \int_0^{\frac{2\pi}{\Omega}} \frac{e^{in\Omega t} dt}{1 - Ga^3}, \qquad (8)$$

where $G = \int_0^\infty q^2 e^{-2qz} r_p(\omega, q)\, dq$ is the tip-sample coupling function and $r_p(\omega, q)$ is the frequency- and momentum-dependent p-polarized reflection coefficient. In the following calculation we concentrate specifically on $n = 5$ as in the main text.

The sample consists of three layers, a TMD top layer, a $30\ nm$ h-BN middle layer, and a thick PPC bottom layer. Due to the large thickness of PPC, it can be safely considered as infinitely thick in the model. The thickness for monolayer TMD is $0.7\ nm$. Therefore, the thickness for the hetero-bilayer case is $1.4\ nm$ and $2.1\ nm$ for the tri-layer case. The dielectric constants of h-BN and PPC are nondispersive in the relevant frequency range and take the values of 3.8 and 3, respectively. The dispersive dielectric function of the TMDs around the exciton resonance frequency can be described by a Lorentz oscillator (eq. 1 in the main text). Once the dielectric function of each layer is known, $r_p(\omega, q)$ can be calculated following the transfer matrix formalism and $s_n e^{i\phi_n}$ can be evaluated using equation (8).

In our case, we aim to solve the reverse problem where $s_n e^{i\phi_n}$ is experimentally measured and the TMD dielectric function is to be solved. This problem can be approached using various techniques[8-11]. Here we employ a fitting procedure: the parameters in equation (1) in the main text are used as free parameters. Staring with estimated initial values for these fitting parameters, iterative optimization algorithms such as the BFGS or the Powell methods are then used to minimize the error function, which is defined as the sum of squared differences, in the parameter space. The parameters that give the best fit are considered as the extracted value and the corresponding $\varepsilon(\omega)$ for the TMD is calculated using equation 1 in main text.

We note that although the point-dipole model might seem like an oversimplification of the realistic tip geometry, its validity has been demonstrated in numerous studies[3,12,13]. Recent simulation study has also demonstrated that the radiation pattern from a realistic tip geometry is very reminiscent of the point-dipole located at the tip apex[5]. Furthermore, we have implemented other models such as the finite dipole model where the elongated tip shank is taken into consideration[14,15]. The conclusion is largely similar. Therefore, we believe the point-dipole model provides sufficient quantitative description of our TMD system in the near-infrared and visible spectral range.

Supplementary Figures

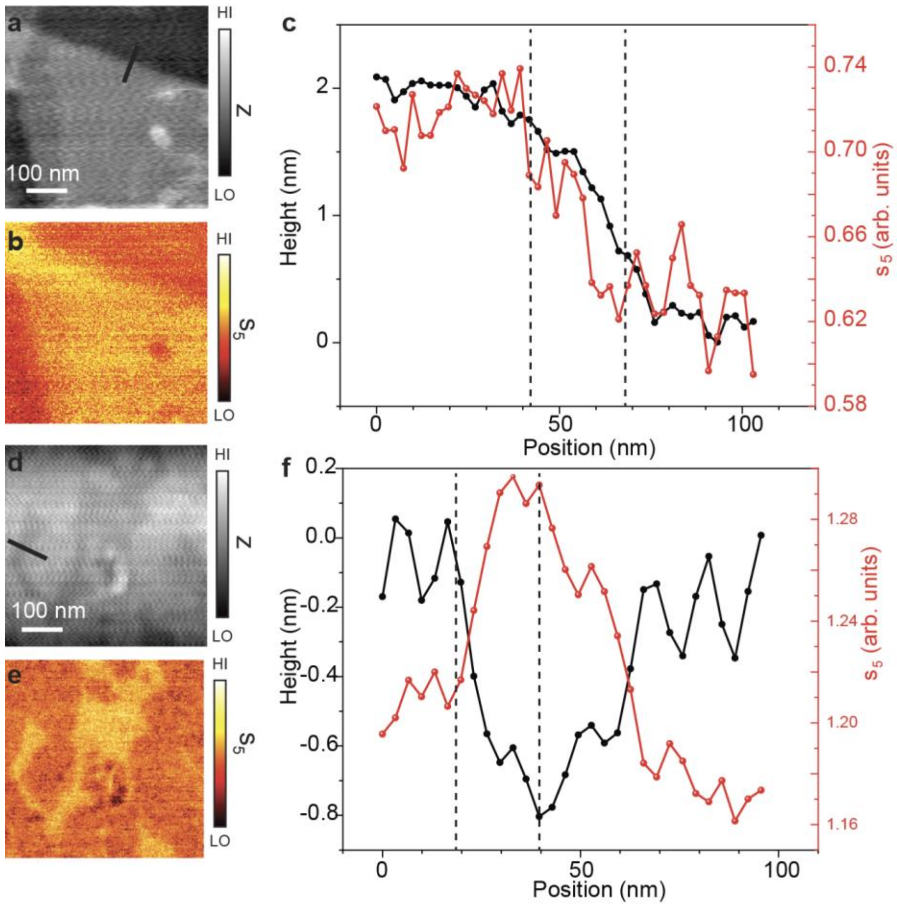

Supplementary Figure 1 | The resolution of the near-field signals. a, AFM topography of WSe_2 trilayer on h-BN. **b,** Near-field images of the amplitude s_5 taken at the excitation energy of 1.66 eV. **c,** Height profile and the near-field amplitude s_5 along the line trace shown in the AFM topography **a**. **d,** AFM topography of close zero degree stacked WSe_2 bilayer on h-BN. **e,** Near-field images of the amplitude s_5 taken at the excitation energy of 1.65 eV. **f,** Height profile and the near-field amplitude s_5 along the line trace shown in the AFM topography **d**. By comparing the topographic images with the near-field images, we can conclude that the resolution of the near-field image is about 20 nm (estimated within a 10% to 90% contrast).

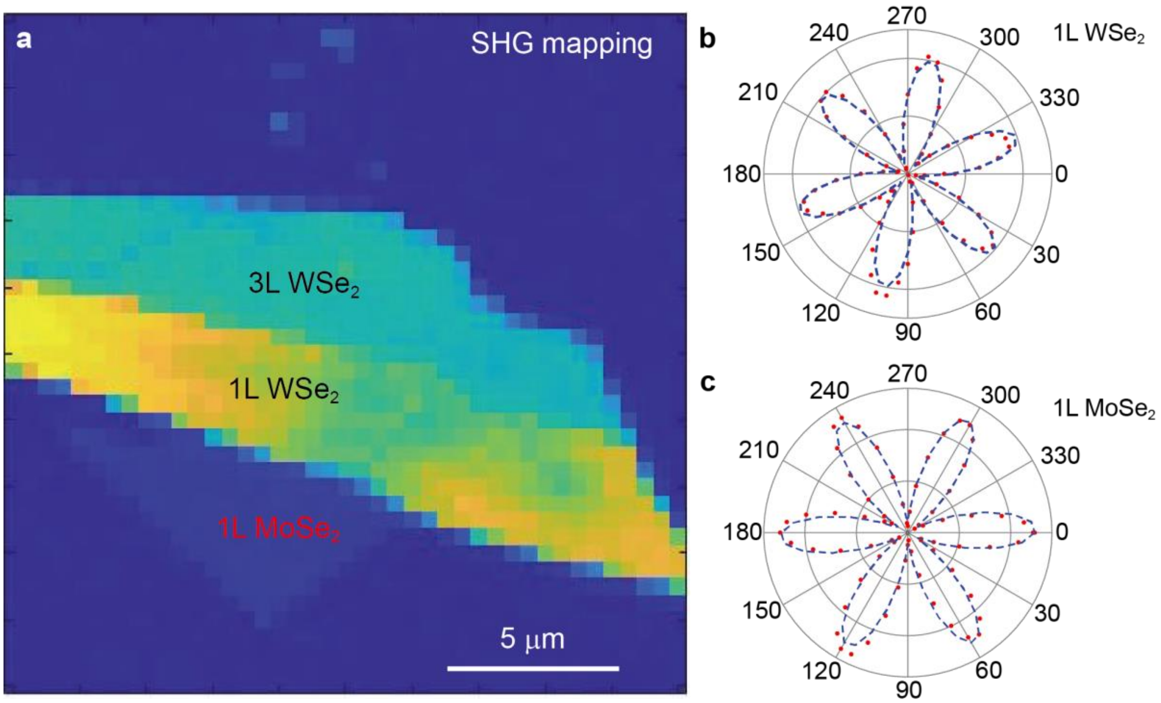

Supplementary Figure 2 | Second harmonic generation (SHG) characterization of the sample. a SHG mapping of the sample. The strong SHG intensity indicates that all the sample regions have odd layer numbers. The inversional symmetry is broken. **b, c** Polarization pattern of SHG intensity of WSe_2 monolayer (**b**) and $MoSe_2$ monolayer (**c**), respectively.

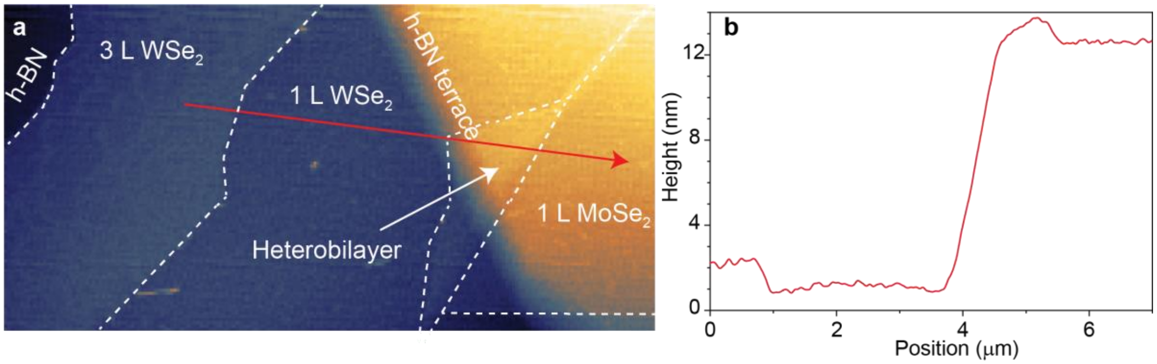

Supplementary Figure 3 | AFM topography. a, AFM topography of the sample region in Fig. 2 in the main text. **b**, Topographic height profile along the red arrow in **a**.

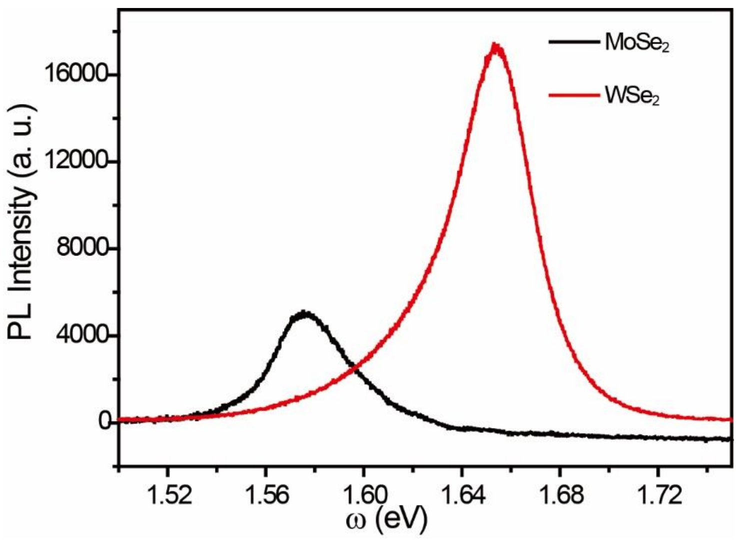

Supplementary Figure 4 | Intralayer exciton photoluminescence (PL) spectra from monolayer $MoSe_2$ and monolayer WSe_2.

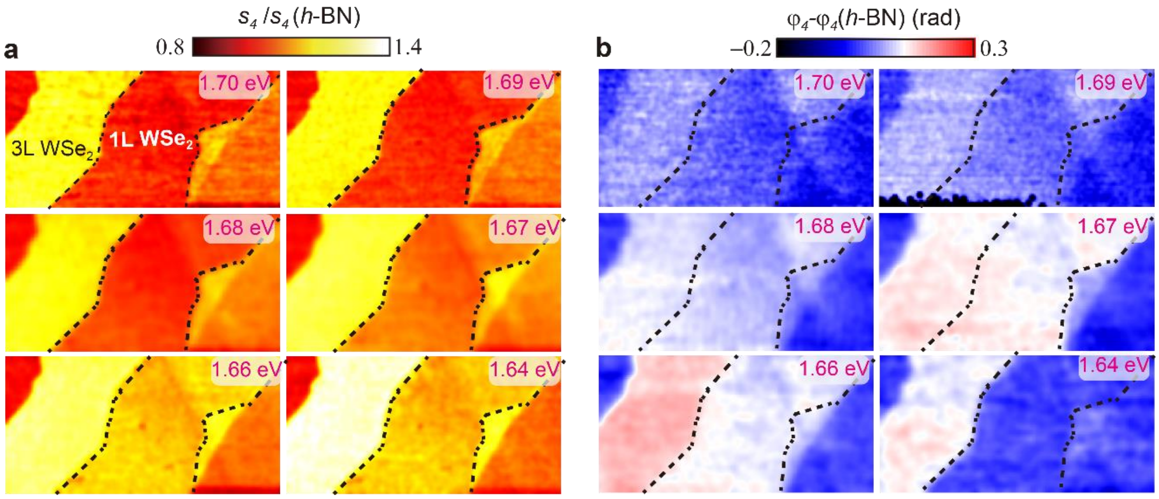

Supplementary Figure 5 | The scattering amplitude and phase demodulated at the fourth harmonic of the tip tapping frequency. a, Representative near-field images of the normalized scattering amplitude. Boundaries of the monolayer WSe_2 region are displayed with black dashed lines. **b**, Near-field images of the normalized phase The excitation energies are indicated in the images. The images are acquired simultaneously with Fig. 2 in main text.

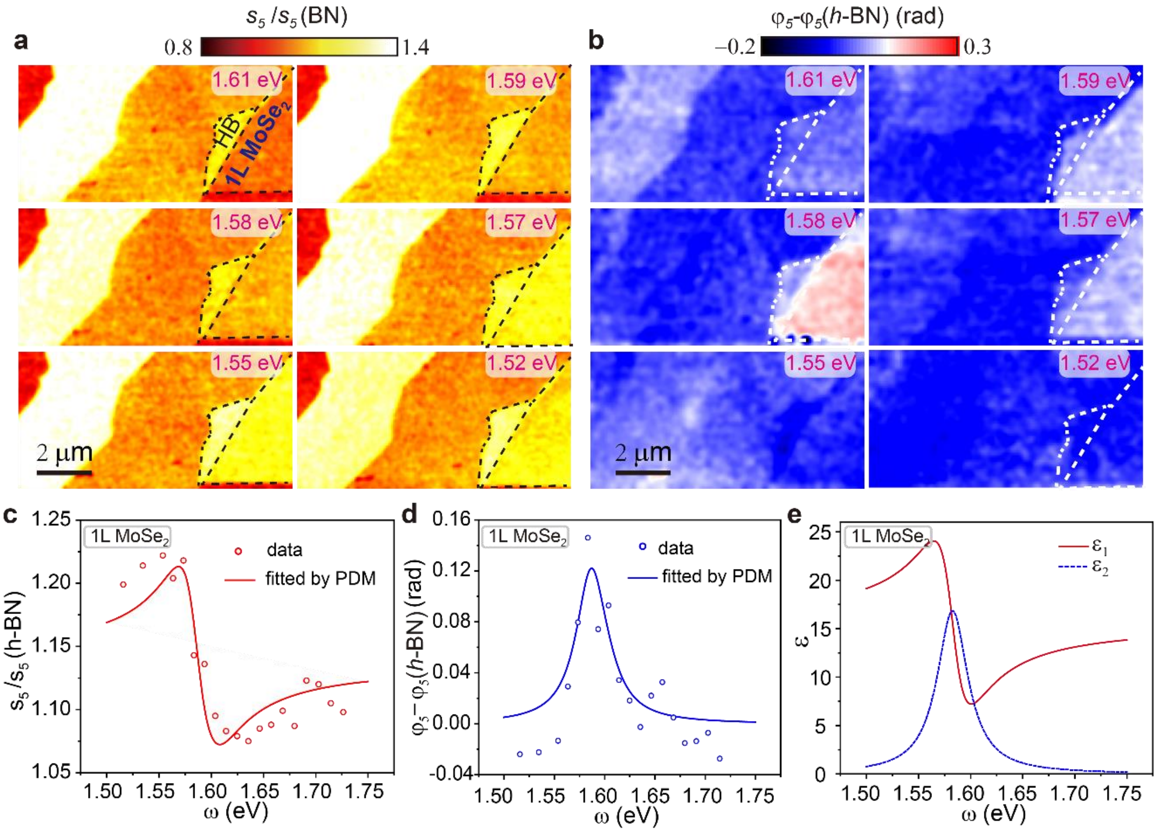

Supplementary Figure 6 | Evolution of the scattering amplitude and phase for the monolayer $MoSe_2$ as a function of the excitation energy. a, Representative near-field images of the normalized scattering amplitude $s_5(\omega)/s_5(h\text{-BN})$. The excitation energies are indicated in the images. The images are acquired by scanning over the rectangular area marked with the dashed lines in Fig. 1 **d**. Boundaries of the monolayer $MoSe_2$ region are displayed with black dashed lines in the upper left panel of **a**. **b**, Near-field images of the normalized phase $\varphi_5(\omega)$. The images with the same energy in **a** and **b** are acquired simultaneously. **c** and **d**, Normalized near-field amplitude $s_5(\omega)/s_5(h\text{-BN})$ and phase $\varphi_5(\omega)$ spectra for $MoSe_2$ (data points). The spectra fitted by the point dipole model (PDM) are shown as solid curves. **e**, Dielectric function of $MoSe_2$ monolayer extracted from the point dipole model.

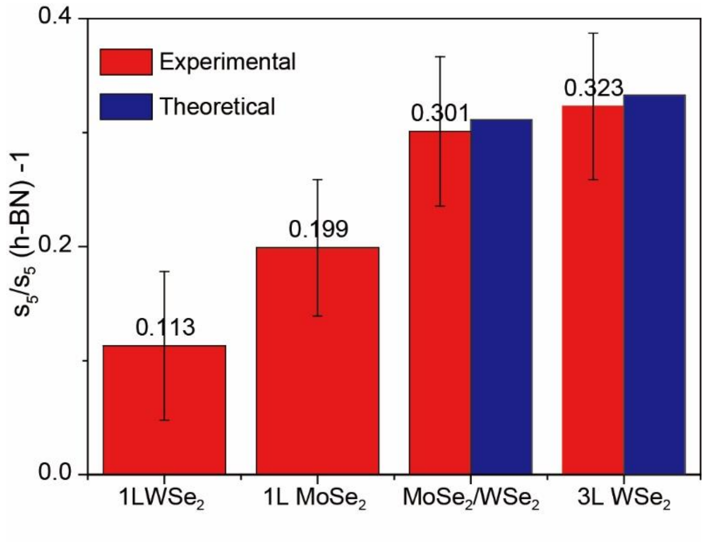

Supplementary Figure 7 | Normalized amplitude dependence on layer numbers. The theoretical values of bilayer and trilayer samples are obtained by adding the signals in monolayers.

Supplementary References

1 Li, Y. *et al.* Measurement of the optical dielectric function of monolayer transition-metal dichalcogenides: MoS2, MoSe2, WS2, WSe2. *Physical Review B* **90**, 205422, doi:10.1103/PhysRevB.90.205422 (2014).

2 Woessner, A. *et al.* Highly confined low-loss plasmons in graphene–boron nitride heterostructures. *Nature Materials* **14**, 421-425, doi:10.1038/nmat4169 (2015).

3 Fei, Z. *et al.* Infrared Nanoscopy of Dirac Plasmons at the Graphene–SiO2 Interface. *Nano Letters* **11**, 4701-4705, doi:10.1021/nl202362d (2011).

4 Zhang, L. M. *et al.* Near-field spectroscopy of silicon dioxide thin films. *Physical Review B* **85**, 075419, doi:10.1103/PhysRevB.85.075419 (2012).

5 Mooshammer, F. *et al.* Quantifying Nanoscale Electromagnetic Fields in Near-Field Microscopy by Fourier Demodulation Analysis. *ACS Photonics* **7**, 344-351, doi:10.1021/acsphotonics.9b01533 (2020).

6 Mester, L., Govyadinov, A. A., Chen, S., Goikoetxea, M. & Hillenbrand, R. Subsurface chemical nanoidentification by nano-FTIR spectroscopy. *Nature Communications* **11**, 3359, doi:10.1038/s41467-020-17034-6 (2020).

7 Hu, D. *et al.* Probing optical anisotropy of nanometer-thin van der waals microcrystals by near-field imaging. *Nature Communications* **8**, 1471, doi:10.1038/s41467-017-01580-7 (2017).

8 Mooshammer, F. *et al.* Nanoscale Near-Field Tomography of Surface States on (Bi0.5Sb0.5)2Te3. *Nano Letters* **18**, 7515-7523, doi:10.1021/acs.nanolett.8b03008 (2018).

9 Govyadinov, A. A. *et al.* Recovery of Permittivity and Depth from Near-Field Data as a Step toward Infrared Nanotomography. *ACS Nano* **8**, 6911-6921, doi:10.1021/nn5016314 (2014).

10 Govyadinov, A. A., Amenabar, I., Huth, F., Carney, P. S. & Hillenbrand, R. Quantitative Measurement of Local Infrared Absorption and Dielectric Function with Tip-Enhanced Near-Field Microscopy. *The Journal of Physical Chemistry Letters* **4**, 1526-1531, doi:10.1021/jz400453r (2013).

11 McLeod, A. S. *et al.* Model for quantitative tip-enhanced spectroscopy and the extraction of nanoscale-resolved optical constants. *Physical Review B* **90**, 085136, doi:10.1103/PhysRevB.90.085136 (2014).

12 Bechtel, H. A., Muller, E. A., Olmon, R. L., Martin, M. C. & Raschke, M. B. Ultrabroadband infrared nanospectroscopic imaging. *Proceedings of the National Academy of Sciences* **111**, 7191, doi:10.1073/pnas.1400502111 (2014).

13 Javier Aizpurua, T. T., F. Javier García de Abajo, Markus Brehm, and Rainer Hillenbrand. Substrate-enhanced infrared near-field spectroscopy. *Opt. Express* **16**, 1529-1545, doi:10.1364/OE.16.001529 (2008).

14 A. Cvitkovic, N. O., and R. Hillenbrand. Analytical model for quantitative prediction of material contrasts in scattering-type near-field optical microscopy. *Opt. Express* **15**, 8550-8565, doi:10.1364/OE.15.008550 (2007).

15 Benedikt Hauer, A. P. E., and Thomas Taubner. Quasi-analytical model for scattering infrared near-field microscopy on layered systems. *Opt. Express* **20**, 13173-13188, doi:10.1364/OE.20.013173 (2012).
